# Supplementary material for: AdpA Positively Regulates Morphological Differentiation and Chloramphenicol Biosynthesis in Streptomyces venezuelae
Source: Microbiol Spectr. 2021 Dec 8;9(3):e01981-21. doi: 10.1128/Spectrum.01981-21 (PMC8653842; doi:10.1128/Spectrum.01981-21)
Supplement: SUPPLEMENTAL FILE 1 — Supplemental material. Download SPECTRUM01981-21_Supp_1_seq6.pdf, PDF file, 0.4 MB [file spectrum01981-21_supp_1_seq6.pdf]

AdpA positively regulates morphological differentiation and chloramphenicol biosynthesis in *Streptomyces venezuelae*

Running title: Role of AdpA regulator in different *Streptomyces*

Małgorzata Płachetka [1], Michał Krawiec [1], Jolanta Zakrzewska-Czerwińska [1] and Marcin Wolański [1]*

* corresponding author, marcin.wolanski@uwr.edu.pl

(1) Faculty of Biotechnology, University of Wrocław, Wrocław, Poland

SUPPLEMENTARY DATA

Supplementary Tables

Table S1. Plasmids and bacterial strains used in this work

Plasmid and strain construction are described in detail elsewhere in Supplementary Materials.

Plasmid	Genotype (description)	Reference / source
p3xFLAG-Myc-CMV-26	Expression dual-tagged vector (N-terminal 3xFLAG an C-terminal *c-myc*)	Sigma-Aldrich
p3xFLAG-Myc-CMV-26-adpA	Derivative of expression plasmid p3xFLAG-Myc-CMV-26 carrying *S. venezuelae adpA* gene cloned into HindIII-BamHI sites.	This work
pET-21a(+)adpAHis$_6$	The expression vector encoding recombinant AdpA of *S. coelicolor*.	(1)
pET-28a(+)	Expression plasmid.	Laboratory stock / (Merck)
pET28-3xFLAG-adpA_Sven	Derivative of expression plasmid pET-28a(+) carrying *3xFLAG-adpA* gene cloned into NheI-BamHI sites.	This work
pAV11b	*Streptomyces* conjugative (*oriT*) and integrative plasmid ($attP_{\varphi BT1}$), pMS82 derivative. Confers hygromycin and tetracycline (Tet^R) resistance (Hyg^R) and contains tetracycline inducible *tcp830* promoter.	(2); courtesy of Dr. Paul Herron
pAVadpA	pAV11b derivative containing copy of $adpA_{Sv}$ gene under control of *tcp830* promoter.	This work
pGEM-T Easy	Cloning vector	Laboratory stock / (Promega)
pMS83	pMS81 with fragment containing tetR from pPC49 cloned in NsiI/KpnI sites; *Streptomyces* conjugative (*oriT*) and integrative plasmid ($attP_{\phi BT1}$) conferring tetracycline resistance	(3)

	(Tet^R).	
pMS83-adpA-3xFLAG	pMS83 derivative containing *$adpA_{Sv}$-3xFLAG* fusion gene expressed from the native *$adpA_{Sv}$* promoter.	This work
PL1-N23	Cosmid carrying *adpA* gene of *S. venezuelae*	Documented at http://strepdb.streptomyces.org.uk
PL1-N23 ΔadpA	PL1-N23 *ΔadpA*::*aac(3)IV* (*adpA* deletion cosmid)	This work
pIJ773	Plasmid template for amplification of the disruption cassette (*aac(3)IV-oriT*) for PCR targeting	(4)
pFLUXH-p0	Integrating (ϕBT1) reporter plasmid with promoterless luciferase operon *luxCDAEB.*	(5, 6)
pTZ57R/T	TA cloning vector conferring ampicillin resistance (Amp^R)	ThermoFisher Scientific
pFLUXH-padpA$_{Sc}$	pFLUXH derivative containing *$adpA_{Sc}$* (SCO2792) promoter region in front of *luxCDAEB*	This work
pFLUXH-padpA$_{Sv}$	pFLUXH derivative containing *$adpA_{Sv}$* (vnz_12630) promoter region in front of *luxCDAEB*	This work
pFLUXH-padpA$_{Sg}$	pFLUXH derivative containing *$adpA_{Sg}$* (SGR_4742) promoter region in front of *luxCDAEB*	This work
pTZ-padpA$_{Sc}$	pTZ57R/T containing *$adpA_{Sc}$* promoter region	This work
pTZ-padpA$_{Sv}$	pTZ57R/T containing *$adpA_{Sv}$* promoter region	This work
pTZ-padpA$_{Sg}$	pTZ57R/T containing *$adpA_{Sg}$* promoter region	This work
pTZ-pdN	pTZ57R/T containing *$pdnaN_{Hp}$* promoter region (negative control lacking AdpA binding consensus)	This work
**Strain**	**Genotype (description)**	**Reference / source**
	Escherichia coli	
DH5α	F^- ϕ80*lac*ZΔM15 Δ(*lac*ZYA-*arg*F)U169 *rec*A1 *end*A1 *hsd*R17(r_K^-, m_K^+) *pho*A *sup*E44 λ^- *thi*-1 *gyr*A96 *rel*A1 λ^-	Laboratory stock (Invitrogen)
E. coli Rosetta™ 2(DE3)	F^-*ompT hsdS*$_B$(r_B^- m_B^-) *gal dcm* (DE3) pRARE2 (Cm^R)	Laboratory stock / (Merck)

ET12567/pUZ8002	*E. coli: dam, dcm, hsdS*, Cam^R, Tet^R containing plasmid pUZ8002: *tra*, Kan^R, *RP4 23*	(7)
E. coli BW25113/pIJ790	Δ(*araD-araB*)*567* Δ*lacZ4787*(::*rrnB4*) *lacIp-40000*(*lacI*^{q}) λ^- *rpoS369*(Am) *rph-1* Δ(*rhaD rhaB*)*568 hsdR514* on the bacterial chromosome; *oriR101 repA1001*(Ts) *araBp-gam-be-exo* on the pIJ790 plasmid	(4)
	Streptomyces venezuelae	
Sven_WT	Wild type *S. venezuelae* NRRL B-65442 strain	(8); John Innes Centre, Norwich UK
Sven_ΔadpA	NRRL B-65442 *adpA::aac(3)IV* (*adpA* deletion mutant)	This work
Sven_ΔadpA/adpA-FLAG	Sven_ΔadpA::pMS83-adpA-3xFLAG (*adpA* deletion mutant complemented with *adpA-FLAG*)	This work
Sven_WT/$adpA^+$	NRRL B-65442::pAVadpA (WT strain with an inducible copy of *adpA*)	This work
Sven_ΔadpA/$adpA^+$	Sven_ΔadpA::pAVadpA (*adpA* deletion mutant complemented with an inducible copy of *adpA*)	This work
Sven_WT_Lux_p0	Sven_WT containing pFLUXH-p0 reporter plasmid	This work
Sven_WT_Lux_$padpA_{Sv}$	Sven_WT containing pFLUXH-$padpA_{Sv}$ reporter plasmid	This work
Sven_WT_Lux_$padpA_{Sc}$	Sven_WT containing pFLUXH-$padpA_{Sc}$ reporter plasmid	This work
Sven_ΔadpA_Lux_p0	Sven_ΔadpA containing pFLUXH-p0 reporter plasmid	This work
Sven_ΔadpA_Lux_$padpA_{Sv}$	Sven_ΔadpA containing pFLUXH-$padpA_{Sv}$ reporter plasmid	This work
Sven_ΔadpA_Lux_$padpA_{Sc}$	Sven_ΔadpA containing pFLUXH-$padpA_{Sc}$ reporter plasmid	This work
	Streptomyces coelicolor	
Sco_WT	*S. coelicolor* A3(2) ($SCP1^-$ $SCP2^-$), wild type strain (also referred to as M145),	(9)
Sco_ΔadpA	M145 *ΔadpA$_{Sc}$* strain (also referred to as M851),	(10)
Sco_WT_lux_p0	M145 containing pFLUXH-p0 reporter	This work

	plasmid	
Sco_WT_lux_padpA$_{Sv}$	M145 containing pFLUXH-padpA$_{Sv}$ reporter plasmid	This work
Sco_WT_lux_padpA$_{Sc}$	M145 containing pFLUXH-padpA$_{Sc}$ reporter plasmid	This work
Sco_WT_lux_padpA$_{Sg}$	M145 containing pFLUXH-padpA$_{Sg}$ reporter plasmid	This work
Sco_ΔadpA_lux_p0	M851 containing pFLUXH-p0 reporter plasmid	This work
Sco_ΔadpA_lux_padpA$_{Sv}$	M851 containing pFLUXH-padpA$_{Sv}$ reporter plasmid	This work
Sco_ΔadpA_lux_padpA$_{Sc}$	M851 containing pFLUXH-padpA$_{Sc}$ reporter plasmid	This work
Sco_ΔadpA_lux_padpA$_{Sg}$	M851 containing pFLUXH-padpA$_{Sg}$ reporter plasmid	This work

Table S2. Oligonucleotides used in this work

**Symbol**	**5' – 3' sequence**	**Description**
	Cloning	
adpA_sven_HindIII_Fd	AAGCTTATGAGCCAGGACTCCGCCGCACCG	Sub-cloning *adpA*$_{Sv}$ gene into p3xFLAG-Myc-CMV-26 and cloning *adpA*$_{Sv}$ gene into pAV11b
adpA_sven_BamHI_Rv	CTGGATCCCTACGGCGCGCTGCGCTGGC	
NheI-FLAG_Fd	GCTAGCGACTACAAAGACCATGACGGTGA	Cloning *adpA*$_{Sv}$ gene into pET-28a(+)
2delta_adpA_sven_Fd	ATCTTCGAGAGCTCCATCCCGCTCTCCGTGTTCGGCATCTTAATTAAATTCCGGGGATCCGTCGACC	Amplification of *aac(3)IV-oriT* cassette, *adpA*$_{Sv}$ disruption
2delta_adpA_sven_Rv	CGACCGAGTAGTCGGAGGTCTCGAGCAGCCGCTGTGCCTTAATTAATGTAGGCTGGAGCTGCTTC	
adpA_sven_prom_region_BglII_Fd	GCAGATCTGGCCCGGCCCACGGGGCG	Cloning *adpA*$_{Sv}$ gene with promoter region into pMS83
adpA_sven_noSTOP_HindIII_PacI	CGTTAATTAACTAAGCTTCGGCGCGCTGCGCTGGCCCG	
3xFLAG_HindIII_Fd	CTAAGCTTGACTACAAAGACCATGACG	Cloning 3xFLAG
3xFLAG_STOP_PacI_Rv	CGTTAATTAATCACTTGTCATCGTCATCCTT	
padpASC-1	TGGATCCTCTAGAATGCATGCGTCTGCTGCTGCGG	Cloning *padpA*$_{Sc}$ into pFLUXH and pTZ vectors
padpASC-2	GAACGAGATCTTCTTCGTCATATGACTGCTAAGCCCCCCTCG	
prom_adpA_EcoRV_Fd	CTGATATCGGCCCGGCCCACGGGGC	Cloning *padpA*$_{Sv}$ into pFLUXH and pTZ vectors
prom_adpA_BamHI_Rv	CTGGATCCGACGCTAAGCCCCCCTCG	
padpASG-2	GAACGAGATCTTCTTCGTCATATGGACGCTAAGCCCCCCT	Cloning *padpA*$_{Sg}$ into pFLUXH and pTZ vectors
padpASG-1	TGGATCCTCTAGAATGCATGCGCCCCGCGACG	

hp0500_BamHI_Rv	CTGGATCCGACTTCTATACTCACTTTGGG	Cloning $pdnaN_{Hp}$ into pTZ vector
hp0500_EcoRV_Fd	CTGATATCATGAAAATCAGTGTTAGTAAAAAC	
qPCR (gene expression)		
qPCRadpASV_fw	CCGGAATCGATCTGTGTCTG	$adpA_{Sv}$ (vnz_12630) expression
qPCRadpASV_rv	GCCGATCTCCTCCGGTAAA	
hrdB-F-sv	GCCGAGTCCGAGTCTGTGA	$hrdB_{Sv}$ (vnz_27210) expression
hrdB-R-sv	CTGGGTTGGCGGAATCTGGT	
qPCR (ChIP samples)		
RT_oriC_AdpAboxes_Fd1	GTCGAGTGAAAGGCGTCAC	origin of replication ($oriC_{Sv}$) region
RT_oriC_AdpAboxes_Rv1	GTTCTGCACACCCGATACAC	
RT_AdpA_prom_Fd1	GGCCATCGGACGGAAGAC	$padpA_{Sv}$ region
RT_AdpA_prom_Rv1	CGGTACCCATGATCGAATCT	
RT-ftsZ-Fw2	GCAGCACCGCAGAACTACCT	$ftsZ_{Sv}$ (vnz_08520) gene
RT-ftsZ-Rv2	GATCATTCGGTTGATGGCATT	
EMSA		
pTZCy5	(Cy5)-TCGGTACCTCGCGAATGCATC	Cy5_padpASv fragment
prom_adpA_BamHI_Rv	CTGGATCCGACGCTAAGCCCCCCTCG	
pTZCy5	(Cy5)-TCGGTACCTCGCGAATGCATC	Cy5_padpASc fragment
padpASC-1	TGGATCCTCTAGAATGCATGCGTCTGCTGCTGCGG	
pTZCy5	(Cy5)-TCGGTACCTCGCGAATGCATC	Cy5_padpASg fragment
padpASG-1	TGGATCCTCTAGAATGCATGCGCCCCGCGACG	
pTZCy5	(Cy5)-TCGGTACCTCGCGAATGCATC	Cy5_HP0500 dnaN (control fragment, lacking AdpASg binding consensus)
hp0500_EcoRV_Fd	CTGATATCATGAAAATCAGTGTTAGTAAAAAC	

Table S3. AdpA boxes in the gene promoter regions within the chloramphenicol gene cluster. Please note that only AdpA boxes with 1 nucleotide mismatches were identified using Pattern Locator. Two equivalent gene notations, “SVEN” and “vnz” were used for the ease of the readers. To view organization of genes within chloramphenicol gene cluster please see: http://strepdb.streptomyces.org.uk/ and (11).

**AdpA binding site sequence**	**Gene** (alternative names)	**Gene product function**
GAACCCGCCG	vnz_04370 (SVEN_0906)	3-oxoacyl-[acyl-carrier protein] reductase
GGGCGGGTTC	vnz_04380 (SVEN_0908)	hypothetical protein
ATTCAGGACA	vnz_04395–vnz_04400 (intergenic) (SVEN_0911–SVEN_0913*; *cmlR*)	hypothetical protein – transcriptional regulator protein
TCGCGGGATC	vnz_04410–vnz_04415 (intergenic) (SVEN_0915; *cmlN*–SVEN_0916; *cmlF*)	sodium/hydrogen exchanger – chloramphenicol efflux pump
GAACCGGACA	vnz_04455–vnz_04460 (intergenic) (SVEN_0924; *cmlI*–SVEN_0925, *cmlM*)	hypothetical protein – hypothetical protein

* please note that due to differences in nucleotide sequences and the genes annotation and organization between “vnz” and “SVEN” sequencing projects is different. In “vnz” annotation SVEN_0912 gene is missing, thus vnz_04395- vnz_04400 genes correspond to SVEN_0911-SVEN_0913. For further details please visit StrepDB and MIBiG database (link below). https://mibig.secondarymetabolites.org/repository/BGC0000893/index.html#r1c1

Supplementary materials and methods

Plasmid construction

pET28-3xFLAG-adpA_Sven

For purification of recombinant *S. venezuelae* AdpA protein, *adpA*$_{Sv}$ gene (vnz_12630) was amplified from *S. venezuelae* chromosomal DNA with appropriate primers (adpA_sven_HindIII_Fd and adpA_sven_BamHI_Rv) and ligated into HindIII- and BamHI-digested p3xFLAG-Myc-CMV-26 plasmid generating p3xFLAG-Myc-CMV-26-adpA. This plasmid was then used as a template for amplification of the *adpA* gene with *3xFLAG* tag using NheI-FLAG_Fd and adpA_sven_BamHI_Rv primers. The resulting PCR product was subsequently cloned into NheI- and BamHI-digested pET-28a(+) vector generating pET28-3xFLAG-adpA_Sven.

pMS83-adpA-3xFLAG

The vector pMS83-adpA-3xFLAG was generated to allow construction of *S. venezuelae* expressing FLAG-tagged fusion AdpA protein used for immunoprecipitation experiments. 3xFLAG tag was amplified from p3xFLAG-Myc-CMV-26 using 3xFLAG_HindIII_Fd and 3xFLAG_STOP_PacI_Rv primers. *S. venezuelae adpA* gene together with the upstream region (459 bp) containing *adpA* promoter was produced using PCR on *S. venezuelae* chromosomal DNA with appropriate primers (adpA_sven_prom_region_BglII_Fd and adpA_sven_noSTOP_HindIII_PacI). The *adpA* gene and promoter (*padpA*) encoding PCR fragment was subcloned into pGEM-T-easy vector. After sequence verification the obtained construct was digested using HindIII and PacI, and ligated with the 3xFLAG encoding HindIII and PacI-digested PCR fragment. Upon sequence verification the entire *padpA-adpA-3xFLAG* region was re-cloned using BglII and PacI restriction enzymes into BamHI (partially) and PacI-digested pMS83. The resulting pMS83-adpA-3xFLAG plasmid was introduced into *S. venezuelae* using *E. coli* conjugative strain.

pAVadpA

adpA$_{Sv}$ gene (vnz_12630) was PCR amplified from *S. venezuelae c*hromosomal DNA with appropriate primers (adpA_sven_HindIII_Fd and adpA_sven_BamHI_Rv). The PCR product was purified and directly cloned into EcoRV-digested and dephosphorylated pAV11b vector. The resulting pAVadpA plasmid was verified using sequencing.

pFLUXH derivatives for luciferase promoter activity assay

DNA fragments containing potential promoter regions were PCR amplified using primers listed in Table S2 and cloned into the reporter plasmid pFLUXH, as described below. All plasmids were verified using restriction digestion and DNA sequencing.

pFLUXH-padpA$_{Sc}$ – *adpA* promoter region of *S. coelicolor* (*padpA*$_{Sc}$) was PCR amplified using padpASC-1 and padpASC-2 primers. The resulting PCR product (500-bp) was purified and directly cloned into Acc65I- and NdeI-digested pFLUXH vector using sequence and ligation-independent ligation (SLIC) (12). Chemically competent *E. coli* DH5α were directly transformed with the SLIC reaction mixture.

pFLUXH-padpA$_{Sv}$ – *adpA* promoter region of *S. venezuelae* (*padpA*$_{Sv}$) was PCR amplified using prom_adpA_EcoRV_Fd and prom_adpA_BamHI_Rv primers. The 459-bp PCR product was EcoRV-and BamHI-digested, the BamHI end was filled-in using Quick Blunting Kit (New England Biolabs), and the fragment was cloned into NdeI-digested pFLUXH and filled-in vector. The reaction mixture was transformed into *E. coli* DH5α cells.

pFLUXH-padpA$_{Sg}$ – *adpA* promoter region of *S. griseus* (*padpA$_{Sg}$*) was PCR amplified using padpASG-1 and padpASG-2 primers. The resulting PCR product (500-bp) was purified and directly cloned into Acc65I- and NdeI-digested pFLUXH vector using sequence and ligation-independent ligation (SLIC) (12). Chemically competent *E. coli* DH5α were directly transformed with the SLIC reaction mixture.

pTZ vectors for amplification of fluorescently labelled DNA fragments

DNA fragments containing potential promoter and control regions were PCR amplified using primers listed in Table S1 and TA cloned into the pTZ57R/T. All plasmids were verified using restriction digestion and DNA sequencing.

pTZ-padpA$_{Sc}$ – *adpA* promoter region of *S. coelicolor* (*padpA$_{Sc}$*) was PCR amplified using *Taq* polymerase and padpASC-1 and padpASC-2 primers. The resulting PCR product (500-bp) was purified and directly cloned into the cloning site of pTZ57R/T vector. Chemically competent *E. coli* DH5α were directly transformed with the reaction mixture.

pTZ-padpA$_{Sv}$ – *adpA* promoter region of *S. venezuelae* (*padpA$_{Sv}$*) was PCR amplified using *Taq* polymerase and prom_adpA_EcoRV_Fd and prom_adpA_BamHI_Rv primers. The 459-bp PCR product was purified and directly cloned into the cloning site of pTZ57R/T vector. Chemically competent *E. coli* DH5α were directly transformed with the reaction mixture.

pTZ-padpA$_{Sg}$ – *adpA* promoter region of *S. griseus* (*padpA$_{Sg}$*) was PCR amplified using *Taq* polymerase and padpASG-1 and padpASG-2 primers. The resulting PCR product (500-bp) was purified and directly cloned into the cloning site of pTZ57R/T vector. Chemically competent *E. coli* DH5α were directly transformed with the reaction mixture.

pTZ-padpA$_{Sg}$ – *adpA* promoter region of *S. griseus* (*padpA$_{Sg}$*) was PCR amplified using *Taq* polymerase and padpASG-1 and padpASG-2 primers. The resulting PCR product (500-bp) was purified and directly cloned into the cloning site of pTZ57R/T vector. Chemically competent *E. coli* DH5α were directly transformed with the reaction mixture.

pTZ-pdN – *dnaN* promoter region of *Helicobacter pylori* (*pdnaN$_{Hp}$*) was PCR amplified using *Taq* polymerase and hp0500_BamHI_Rv and hp0500_EcoRV_Fd primers. The resulting PCR product (403-bp) was purified and directly cloned into the cloning site of pTZ57R/T vector. Chemically competent *E. coli* DH5α were directly transformed with the reaction mixture.

Construction of *Streptomyces* strains

Generation of adpA mutant strain of S. venezuelae and its derivatives

The *adpA* deletion mutant (Sven_ΔadpA) was obtained using the PCR targeting strategy (4). To replace the *adpA$_{Sv}$* coding region within PL1-N23 cosmid with an apramycin resistance cassette (*aac(3)IV-oriT*), the apramycin cassette was PCR amplified from pIJ773 plasmid with appropriate primers (2delta_adpA_sven_Fd and 2delta_adpA_sven_Rv). The resulting PCR product was used to transform BW25113 carrying PL1-N23 cosmid and λ RED plasmid, pIJ790. The obtained recombinant cosmid PL1-N23 ΔadpA was verified using restriction digestion and PCR and subsequently used for conjugation of *S. venezuelae* NRRL B-65442 with *E. coli* ET12567(pUZ8002) carrying this cosmid. Following selection for the single-crossover exconjugants exhibiting Apr^R (apramycin resistance) carrying PL1-N23 ΔadpA integrated in the *S. venezuelae* chromosome, the transconjugants were subsequently screened for the loss of Kan^R, (kanamycin resistance) indicating a double-crossover allelic

exchange of the *adpA* locus.

Two approaches were used to obtain chromosome integrative *adpA* complementation. In the first one, the $adpA_{Sv}$ gene was introduced into the Sven_ΔadpA using pAVadpA plasmid. The resulting strain Sven_ΔadpA/adpA^{+} allows controlled expression of $adpA_{Sv}$ placed under anhydrotetracycline inducible *tcp830* promoter. In the second approach, the $adpA_{Sv}$*-3xFLAG* fusion gene under the control of native $adpA_{Sv}$ promoter was delivered into the *adpA* deletion mutant strain using pMS83-adpA-3xFLAG, resulting in Sven_ΔadpA/adpA-FLAG. In both cases the plasmids were introduced via conjugation with the *E. coli* ET12567(pUZ8002) carrying respective integrative plasmid in the Sven_ΔadpA.

Strains for luciferase promoter activity assays

The pFLUXH plasmid and its derivatives containing promoter regions of *adpA* genes were introduced into *S. venezuelae* and *S. coelicolor* strains via conjugation.

Recombinant protein expression and purification

The AdpA protein of *S. venezuelae* was purified as a recombinant N-terminally His-tagged AdpA (6xHis-3xFlag-AdpA, referred also as AdpASv_His) from heterologous *E. coli* Rosetta™ 2(DE3) (Merck) strain harboring the pET28a-3xFLAG-adpA_Sven plasmid (for plasmid construction details, see in the SI). To produce AdpASv_His protein an overnight starting culture in Terrific Broth (TB) medium (Cold Spring Protocols, recipe no. 8620) supplemented with antibiotics (kanamycin 100 µg/ml and chloramphenicol 34 µg/ml) was diluted at 1/5 ratio with a fresh portion of TB medium (kanamycin 100 µg/ml). The culture broth (800 ml in 2.8-liter baffled flask) was then incubated in a shaker incubator (180 rpm, 37°C, 7 hours). The cultures were subsequently transferred to 20°C and supplemented with 5xTB concentrate (80 ml per 800 ml of culture broth), and the IPTG (1 mM) (Isopropyl β-D-1-thiogalactopyranoside) was added to induce protein expression. Following the induction cultures were incubated overnight at 20°C and harvested using centrifugation, the cell pellets were stored at -20°C. The protein purification was performed from 1.6-litre of culture. At the time of use the cell pellets were thawed on ice and resuspended in lysis buffer A (50 mM NaH_2PO_4, 300 mM NaCl, 10 mM imidazole, pH 8.0) (5 ml of buffer per 1 g of cell paste) supplemented with protease inhibitors (A32965, ThermoFisher Scientific) and universal nuclease (88702, ThermoFisher Scientific). The cells were were disrupted by sonication (Sonics) (5s ON/ 5s OFF, amplitude 40-50%, 10 minutes sonication time) and clarified by centrifugation (45 min., 37 000 x g, 4°C). The AdpASv_His protein was purified on HiTrap Talon® crude column (1 ml, GE Healthcare) using Äkta start system (GE Healthcare) according to system built-in column protocol with minor modifications. The protein was eluted from the column using a gradient of elution buffer B (50 mM NaH_2PO_4, 300 mM NaCl, 500 mM imidazole, pH 8.0) in lysis buffer A. The protein fractions were examined using sodium dodecyl sulfate-polyacrylamide gel electrophoresis (SDS-PAGE), similar fractions were pooled, aliquoted and flash frozen with liquid nitrogen. The protein was stored at -80°C.

The recombinant AdpA protein of *S. coelicolor* (AdpASc_His) was purified with the above procedure using *E. coli* Rosetta™ 2(DE3) harboring the pET-21a(+)adpAHis$_6$ plasmid (1).

ChIP-qPCR (Chromatin Immunoprecipitation-quantitative PCR)

ChIP

To study AdpA binding on *S. venezuelae* chromosome a chromatin immunoprecipitation (ChIP) assay

(13) followed by qPCR was performed The Sven_WT and Sven_ΔadpA/adpA-FLAG (*ΔadpA* complementation) strains (two independent cultures) were grown in the MYM medium supplemented with trace elements for an appropriate time (12 and 20 h). After crosslinking (formaldehyde added to a culture at final concentration of 1% v/v) and cell lysis, samples were sonicated for 3-4 cycles (15 s ON/15 s OFF, 15 s ON/15 s OFF, amplitude 40%) to shear the chromosome into fragments ranging from 200 to 1000 bps. The samples (1.5 ml) were centrifuged twice (16000 x g, 4°C for 15 minutes), and the supernatants were incubated for 3 h at 4°C with 30 µl of Anti-FLAG M2 Magnetic Beads (cat. no. M8823, Sigma-Aldrich) previously pre-equilibrated with IP buffer (50 mM Tris-HCl pH 8.0, 250 mM NaCl, 0.80% Triton, protease inhibitors (cat. no A32955, ThermoFisher Scientific). After incubation the magnetic beads were washed twice with IP_500 buffer (IP buffer with 500 mM NaCl) to remove non-specifically bound protein-DNA complexes and further incubated overnight at 65°C in TE buffer (50 mM Tris-HCl pH 8.0, 10 mM EDTA) supplemented with 1% SDS for decrosslinking. The samples were then incubated with proteinase K (Roche) for 1.5 h at 55ºC and extracted with phenol and subsequently with chloroform. The DNA fragments were precipitated with ethanol and sodium acetate using standard protocol. DNA concentration was quantified using a NanoDrop spectrophotometer (Thermo Fisher Scientific).

qPCR

For each immunoprecipitation sample the qPCR experiments were performed to assess quantity of target DNA fragments using primers listed in Table S2. The qPCR analyses were conducted using StepOnePlus system (Applied Biosystems) and PowerUp SYBR Green Master Mix (ThermoFisher Scientific). The qPCR experiments were performed using ~2 ng DNA and the following thermocycling conditions: 95°C for 10 minutes, followed by 40 cycles of 30 seconds at 95°C, 30 seconds at 60°C and 30 seconds at 72°C. The specificity of each primer pair was confirmed by performing melt curve analysis included in the qPCR protocol according to default protocol included in StepOnePlus system for SYBR green detection. Performance of each primer pair was analyzed using StepOne Software v 2.0 (Applied Biosystems). The cycle threshold (Ct) values obtained for target DNA fragments were used to manually calculate, using ΔΔCt method, relative quantities (RQ) of corresponding DNA fragments (GOI – gene of interest) internally normalized to the level of *ftsZ* gene fragment (vnz_08520) (normalizer). The average RQ value of two biological samples was referred to IP samples derived from Sven_WT collected at 12 h (calibrator strain). Experiment was performed in biological duplicate (two independent IP isolates) and each sample was conducted in technical triplicate.

SDS-PAGE and Western Blot

Sven_ΔadpA/adpA-FLAG strain cell lysates were prepared from liquid cultures (two replicates) in the MYM medium sampled every two hours. For sodium dodecyl sulphate-polyacrylamide gel electrophoresis (SDS-PAGE) (14) samples of frozen mycelium were resuspended in PBS buffer (137 mM NaCl, 2.7 mM KCl, 10 mM Na_2HPO_4, 1.8 mM KH_2PO_4) supplemented with protease inhibitors (cat. no A32955, ThermoFisher Scientific) and sonicated for 3-4 cycles (15 s ON/15 s OFF, amplitude 40%). Total protein concentration was determined using the Bradford assay (Carl-Roth). 40 µg of total protein was loaded into each gel lane. Following electrophoresis, the proteins were transferred onto the nitrocellulose membrane (Amersham™ Protran™ 0.45 µm NC, GE Healthcare Life Science). Upon transfer the membrane was blocked using skim milk dissolved (3% w/v) in TBST buffer (10 mM

Tris-HCl pH 8.0, 150 mM NaCl, 0.05% Tween 20). The membrane was subsequently incubated with primary mouse anti-FLAG antibody (F3165, Sigma-Aldrich) and secondary goat anti-mouse antibody fused with horse radish peroxidase (cat. no. sc-2005, Santa Cruz Biotechnology). The protein was visualized using SuperSignal™ West Pico PLUS Chemiluminescent Substrate (ThermoFisher Scientific) according to manufacturer's instructions. The images were recorded using ChemiDoc MP system (Bio-Rad).

Electrophoretic mobility shift assay (EMSA)

For the analysis of protein-DNA interaction using EMSA, the AdpASc_His and AdpASv_His recombinant proteins and the fluorescently labelled DNA fragments were used as described below. Fluorescently labelled with the Cy5 dye DNA fragments were prepared by PCR amplification using Q5 High-Fidelity Polymerase (New England Biolabs), primers listed in Table S2 and vector templates listed in Table S1. To confirm uniformity and specificity the PCR samples were then run on the agarose gels and subsequently purified of the unincorporated primers and nucleotides using exonuclease I (ExoI) and alkaline phosphatase (FastAP) enzyme mixture (ThermoFisher Scientific) according to a manufacturer's recommendations followed by purification using standard silica column kit. The concentration of the DNA was assessed using NanoDrop 1000 (ThermoFisher Scientific). The Cy5-labelled DNA fragments (20 ng per sample) were incubated with increasing amounts of recombinant proteins in the presence of BSA (5 µg/µl) and poly(dI-dC)·poly(dI-dC) (3.75 ng/µl) competitors in HBS200 buffer (10 mM HEPES pH 7.4, 10 mM MgOAc, 200 mM NaCl, 3.4 mM EDTA, 0.05% Tween 20, 5% glycerol) for 30 minutes at 25°C. Before sample application the gel was pre-run for 30 min at 100 V 4-8°C. The samples were then resolved in 15 cm long, 5% native polyacrylamide gels (37.5:1, 3029.2 Carl Roth) prepared in 0.25x TBE buffer and run in the same buffer at 100 V for about 4 hours at 4-8°C. Upon completion of the electrophoresis the gels were analyzed using the ChemiDocMP System (Bio-Rad).

Gene expression (RNA isolation and RT-qPCR)

RNA isolation

The total RNAs were isolated from *S. venezuelae* liquid cultures grown for 24 hours at 30°C in MYM medium (without antibiotics) supplemented with trace element solution. Samples were collected at two-hour intervals between 14 and 22 hours. Each culture suspension (1.5 ml) was immediately mixed in 2-ml Eppendorf tube with 1/5 volume (0.3 ml) of the Stop Solution (95% ethanol plus 5% in water saturated phenol pH 4.5-5) (Jahn et al. 2008) and centrifuged (15000 x g, room temperature, 30 seconds). The supernatant was discarded, and the cell pellet was flash frozen in liquid nitrogen and stored at -80°C. At the time of use, 1.8 ml of TRI Reagent solution (ThermoFisher Scientific) was added to a frozen pellet and the cells were resuspended by pipetting. Equal volumes of the cell suspension (~ 1 ml) was then transferred into two 1.5-ml screw-cap tubes containing 0.4 ml of zirconia/silica beads (0.1-mm) and placed into FastPrep-24 homogenizer (MP Biomedical) for 2 cycles (45 s, speed 6 m/s) with 3 min of cooling on ice between cycles. Samples were centrifuged (15000 x g, 4°C for 5 min) and the supernatants transferred to fresh tubes containing 0.2 ml chloroform. Upon mixing for 30s by inversion, the solution was left at room temperature for 10 min and subsequently centrifuged (15000 x g, 4°C for 15 min). The upper water phase (~250 ml) was transferred into a fresh tube and mixed with isopropanol (450 ml). The resulting solution was

applied onto the silica column and purified according to manufacturer's instructions (Total RNA Mini Kit, A&A Biotechnology). RNA concentration was quantified using a NanoDrop ND-1000 spectrophotometer, and the quality of the RNA was analyzed on an agarose gel.

RT-qPCR

cDNA was obtained by reverse transcription of 1–4 µg of DNase I-treated total RNA using Maxima First Strand cDNA Synthesis Kit for RT-qPCR (ThermoFisher Scientific) following procedures recommended by the manufacturer. Gene expression was analyzed by qPCR using a StepOnePlus system (Applied Biosystems) and PowerUp SYBR Green Master Mix (ThermoFisher Scientific). qPCR was performed using 50–300 ng cDNA and the following thermocycling conditions: 95°C for 10 minutes, followed by 40 cycles of 30 seconds at 95°C, 30 seconds at 60°C and 30 seconds at 72°C. The primers used for qPCR (Table S2), were designed using Primer-BLAST tool at NCBI. The specificity of each primer pair was confirmed by performing melt curve analysis included in the qPCR protocol according to default protocol included in StepOnePlus system for SYBR green detection. Performance of each primer pair was analyzed using StepOne Software v 2.0 (Applied Biosystems). Transcript levels of target genes were normalized internally to the level of the RNA polymerase sigma factor *hrdB* homolog (vnz_27210) gene as in previous studies (15, 16). Each experiment was performed in three biological replicates, and each sample was measured in three technical replicates. With each set of primers, negative-control experiments, performed in the absence of reverse transcriptase, confirmed that PCR products were amplified from a cDNA template (i.e., there was no significant contamination with chromosomal DNA).

Disc diffusion assays to assessment antibacterial activity

Chloramphenicol activity against indicator strains

For assessment of chloramphenicol production in liquid cultures the GYM medium (per 1 liter: malt extract 10 g, yeast extract 4 g, glucose 4 g, CaCl2 1.46 g, pH 7.3) was used. The 250-ml baffled flask containing 50 ml of the medium was inoculated with the spore suspension of either wild-type or *adpA* deletion *S. venezuelae* strain (Sven_WT or Sven_ΔadpA, respectively) to reach the final spore OD of 0.002. The culture was grown for 3 days in a rotary shaker incubator (30°C, 200 rpm). At the indicated time point the entire culture broth (50 ml) was centrifuged (10 min at 5000 g, room temperature) and the supernatant was immediately extracted with an equal volume of ethyl acetate. After shaking for 30 min, the extract was centrifuged, and the organic phase aliquoted to 15-ml falcon-type tubes. The extracts were pre-concentrated under reduced pressure using rotary evaporator to approximately 1/10 of the initial volume, then the samples from the same culture flask were pooled into one tube and continued with the concentration procedure. The solid residues were re-dissolved in 150 µl of a methanol:water mixture (2:3). To prepare the discs for antibiotic resistance test a 50 µl of each re-dissolved extract was spotted onto a sterile paper disc and dried for 5 min under laminar hood. The control discs contained either the methanol:water or 30 µg of commercially derived chloramphenicol dissolved in methanol:water solution. To assess chloramphenicol production the extracts were prepared from three independent broth cultures. The antibiotic activities of the extracts were determined using modified Kirby-Bauer agar diffusion method. The discs soaked with corresponding solutions were applied onto the surface of the solid LB medium inoculated with the appropriate chloramphenicol sensitive (*Micrococcus luteus* or *Escherichia coli* DH5α) or chloramphenicol resistant (*Escherichia coli* ET12567) indicator strain and

incubated for 24 h at 37°C.

Chloramphenicol titer

To estimate concentration of chloramphenicol in culture broth extracts the diameters of inhibition zones around the discs soaked with the extracts were measured and plotted against the standard curve generated using discs soaked with defined amounts of chloramphenicol. The *M. luteus* was used as the indicator strain.

Bioinformatics analyses

The Ugene software was used to make sequence alignments (17). The graphical shading of amino acid alignment result files was performed using on-line BOXSHADE 3.21 software at https://embnet.vital-it.ch/software/BOX_form.html.

Phylogenetic relationships between amino acid (AdpA proteins) and nucleotide sequences (16S RNA genes) have been performed using One Click Workflow (https://ngphylogeny.fr/) (18, 19) and iTOL (https://itol.embl.de/) (20). The amino acid and nucleotide sequences have been derived from Streptomyces database at http://streptomyces.org.uk/.

In silico identification of AdpA boxes was performed using Pattern Locator tool (http://www.cmbl.uga.edu/software/patloc.html) (20). The searches were conducted on both strands using default software parameters.

Figures

Fig. S1. Sequence alignment of AdpA orthologues.

The amino acid protein sequences are designated by their gene names and listed in the following order: *S. venezuelae*, *S. coelicolor*, *S. griseus*, *S. lividans*, *S. avermitilis*, *S. clavuligerus*, *S. leeuwenhoekii*. The color bars spanning over the sequences indicate: blue – oligomerization domain, pink – DNA-binding domain, yellow – HTH motifs. The short vertical grey bars indicate conserved arginine residues (based on (21, 22)). The alignments and the sequence shadings were generated using Ugene software (ClustalW algorithm) and BOXSHADE tool. The sequences presented in this alignment were derived from http://streptomyces.org.uk.

Fig. S2. Phylogenetic relationships among *Streptomyces* strains based on AdpA protein and 16S rRNA gene sequences.

(A) The phylogenetic analysis of AdpA orthologues. Proteins are designated by their gene names, species names are given in parentheses. **(B)** The phylogenetic analysis 16S rRNA sequences. The corresponding genes names are given together with species names.

The analyses were performed using One Click Workflow tool (https://ngphylogeny.fr/) and the resulting phylogenetic trees were directly exported and processed using iTOL (https://itol.embl.de/). All sequences were derived from *Streptomyces* database at http://streptomyces.org.uk/. In both, panels, the tree scales are given in the legends and branches lengths are given in red numbers.

Figure S3. Gene organization of selected *adpA* loci in *Streptomyces*.

Schematic depictions of gene organization (left panel) and approximate locations of *adpA* loci on chromosomes (right panel) of the corresponding *Streptomyces* species. The *adpA* genes are shown as green arrows; the numbers indicate Mbps. For the convenience, the orientations of *adpA* genes in the left panel are shown in left to right convention – note that the actual orientations of *adpA* genes on the chromosomes are shown in the right panel. The *uspA* and *ornA* genes encode a conserved universal shock protein A and oligoribonuclease, respectively (discussed in the text).

Figure S4. Comparison of growth phenotypes of *S. venezuelae* strains.

(A) Phenotypes of the wild-type Sven_WT (WT), disruption Sven_ΔadpA (delta) and complemented Sven_ΔadpA/adpA-FLAG (compl) strains grown for 6 days on solid ISP-2, GYM, SFM and MYM media. Please note that the photographs were taken on a dark background thus the aerial hyphae may look more as a white surface, and the vegetative mycelium may look grey (e.g. deletion mutant on MYM). **(B)** Phenotypes of *S. venezuelae* strains grown on MYM solid in the presence of anhydrotetracycline (ATET) inducer. The Sven_WT/adpA^{+} (WT/adpA^{+}) and Sven_delta/adpA^{+} (delta/adpA^{+}) strains harbor pAVadpA integration plasmid enabling overexpression of *adpA*$_{Sv}$ gene from *tcp830* promoter upon supplementation the medium with ATET; the Sven_WT and Sven_ΔadpA (designations as in panel A) lack the integration plasmid. The strains were cultivated for 48 hours. ATET concentrations are given in the picture. **(C)** Appearance of the spore suspensions of Sven_WT and Sven_ΔadpA strains. Spores were collected from solid MYM medium. Zoom-in is shown in the small panel. **(D)** Phenotypes of Sven_WT and Sven_ΔadpA strains grown in MYM liquid medium. Cultures were photographed after ~20 hours of cultivation with shaking. Zoom-in is shown in the small panel.

Figure S5. Occurrence of AdpA binding sites within *padpA* and *oriC* regions.

The analyses of the presence of AdpA boxes (A_box) within *padpA* **(A)** and *oriC* **(B)** regions in *S. venezuelae*, *S. coelicolor* and *S. griseus* based on *in silico* search and literature data. The AdpA boxes are shown as white and grey arrows in accordance with their orientations; boxes identified in this study using *in silico* analysis are indicated by asterisks; the numbers in parentheses indicate number of mismatches within the respective sequences in relation to AdpASg consensus binding sequence (5'- TGGCSNGWWY-3') (23). In panel A, the conservative AdpA box is shown in grey and the red arrows marked with TS indicate transcriptional start sites within corresponding promoters (10, 24). TS** - the precise location of the *adpA*$_{Sv}$ promoter was determined as part of a genome-wide 5' triphosphate end-capture transcription start site mapping experiment (M. Bush and M. Buttner, Pers. Comm. also available at http://streptomyces.org.uk/vnz_tss.html)

Figure S6. Purification of recombinant AdpASv_His and AdpASc_His proteins.

SDS-PAGE analysis of AdpASv_His **(A)** and AdpASc_His **(B)** proteins purified using affinity chromatography on HiTrap Talon® crude column. The successive fractions collected during elution with increasing gradient of imidazole (10-250 mM) are indicated by consecutive numbers above the gel lanes. Bands representing proteins of interest are indicated by arrows together with their molecular weights. **(C)** Loading control for western blot analysis shown in Fig. 2B. Equal amounts of Sven_ΔadpA/adpA-FLAG strain cell lysates (40 µg per lane) were loaded in corresponding wells. Numbers above the gel indicate sample collection times. **(D)** Western blot analysis – no-FLAG control. Equal amounts of Sven_WT (no-FLAG control) and Sven_ΔadpA/adpA-FLAG cell lysates (WT and FLAG, respectively) were used to verify the specificity of anti-FLAG antibody. Chemiluminescent and EPI (visible light) signals were recorded for the same blot. SDS-PAGE represents loading control for corresponding samples. The protein marker (MW) (cat. no. 26610, ThermoFisher Scientific) is shown together with molecular weights (kDa) of proteins comprising the ladder.

Figure S7. Activities of *adpA* promoters in native and heterologous hosts.

The time-course analysis of *adpA*$_{Sv}$ and *adpA*$_{Sc}$ promoters activities in *S. venezuelae* **(A)** and *S. coelicolor* **(B)** hosts conducted using luciferase reporter system encoded on pFLUXH integration vector. The strains of *S. venezuelae* and *S. coelicolor* were grown on solid MYM and DNA media, respectively and the measurements were recorded at corresponding time points. The designations lux_padpASc, lux_padpASv and lux_p0 stand for pFLUXH plasmids harboring respective *adpA* promoters (*S. coelicolor* and *S. venezuelae*) or control empty plasmid, respectively. The wild-type (Sven_WT and Sco_WT) and the *adpA* disruption (Sven_ΔadpA and Sco_ΔadpA) strains harboring respective pFLUXH vectors are described in the legends. The bars represent the average of medians for 3 independent clones and 4 technical replicates, respectively; the error bars represent standard deviation. Graphs were generated in Microsoft Excel.

Figure S8. Antibiotic production in *S. venezuelae* strains.

(A) Antibacterial activities of culture broth extracts derived from Sven_ΔadpA (delta) and Sven_ΔadpA/adpA-FLAG (compl) strains against *M. luteus*. The assay was conducted by disc diffusion method using extracts obtained from three independent cultures, here designated by numbers. A control (K) disc was prepared using solvent (methanol:water) solution. **(B)** Sensitivity of *M. luteus* to defined amounts of chloramphenicol. The discs were soaked with designated quantities of antibiotic (µg per disc) and applied onto the *M. luteus* loans in two technical repetitions (tr1 and tr2). The diameters of inhibition zones and antibiotic concentrations are given in yellow. **(C)** Standard curve – dependence of the diameter of inhibition zone on the

chloramphenicol (CHLF) quantity. The graph was based on the measurements presented in panel B. The trend line equation was calculated using Microsoft Excel. **(D)** Assessment of antibacterial activities of Sven_WT culture broth extracts by disc diffusion assay. Three independent cultures (cult.1, cult.2 and cult.3) in two technical repetitions (tr1 and tr2) were tested against *M. luteus*. tested. The diameters of inhibition zones are given in yellow.

In panels **(B)** and **(D)** the diameters of inhibition zones were measured in CorelDRAW Graphics Suite X5.

Figure S9. Genes organization within Cm-BGC.

Schematic depiction of chloramphenicol biosynthetic gene cluster of *S. venezuelae*. Gene annotations from different sequencing projects of *S. venezuelae* NRRL B-65442 are shown below the genes according to StrepDB (http://streptomyces.org.uk/) and previous reports (25). Locations of *in silico* predicted AdpA boxes are indicated using red triangles.

References

1. Wolański M, Donczew R, Kois-Ostrowska A, Masiewicz P, Jakimowicz D, Zakrzewska-Czerwińska J. 2011. The level of AdpA directly affects expression of developmental genes in Streptomyces coelicolor. J Bacteriol 193:6358–6365.
2. Khaleel T, Younger E, Mcewan AR, Varghese AS, Smith MCM. 2011. A phage protein that binds ϕC31 integrase to switch its directionality. Mol Microbiol 80:1450–1463.
3. Gregory MA, Till R, Smith MCM. 2003. Integration site for Streptomyces phage ϕBT1 and development of site-specific integrating vectors. J Bacteriol 185:5320–5323.
4. Gust B, Challis GL, Fowler K, Kieser T, Chater KF. 2003. PCR-targeted Streptomyces gene replacement identifies a protein domain needed for biosynthesis of the sesquiterpene soil odor geosmin. Proc Natl Acad Sci U S A 100:1541–6.
5. Szafran MJ, Gongerowska M, Gutkowski P, Zakrzewska-Czerwińska J, Jakimowicz D. 2016. The coordinated positive regulation of topoisomerase genes maintains topological homeostasis in Streptomyces coelicolor. J Bacteriol 198:3016–3028.
6. Craney A, Hohenauer T, Xu Y, Navani NK, Li Y, Nodwell J. 2007. A synthetic luxCDABE gene cluster optimized for expression in high-GC bacteria. Nucleic Acids Res 35:1–10.
7. Paget MSB, Chamberlin L, Atrih A, Foster SJ, Buttner MJ. 1999. Evidence that the extracytoplasmic function sigma factor σ^E is required for normal cell wall structure in *Streptomyces coelicolor* A3(2). J Bacteriol 181:204–211.
8. Gomez-Escribano JP, Holmes NA, Schlimpert S, Bibb MJ, Chandra G, Wilkinson B, Buttner MJ, Bibb MJ. 2021. Streptomyces venezuelae NRRL B-65442: genome sequence of a model strain used to study morphological differentiation in filamentous actinobacteria. J Ind Microbiol Biotechnol 0:35.
9. Kieser T, Bibb MJ, Buttner MJ, Chater KF, Hopwood DA. 2000. Practical Streptomyces Genetics. A Laboratory Manual. John Innes Foundation, Norwich, United Kingdom.
10. Takano E, Tao M, Long F, Bibb MJ, Wang L, Li W, Buttner MJ, Bibb MJ, Deng ZX, Chater KF. 2003. A rare leucine codon in adpA is implicated in the morphological defect of bldA mutants of Streptomyces coelicolor. Mol Microbiol 50:475–486.
11. Zhang X, Andres SN, Elliot MA. 2021. Interplay between Nucleoid-Associated Proteins and Transcription Factors in Controlling Specialized Metabolism in Streptomyces. MBio 12:1077–1098.
12. Li MZ, Elledge SJ. 2007. Harnessing homologous recombination in vitro to generate recombinant DNA via SLIC. Nat Methods 4:251–256.
13. Al-Bassam MM, Bibb MJ, Bush MJ, Chandra G, Buttner MJ. 2014. Response Regulator Heterodimer Formation Controls a Key Stage in Streptomyces Development. PLoS Genet 10:e1004554.
14. Laemmli UK. 1970. Cleavage of structural proteins during the assembly of the head of bacteriophage T4. Nature 227:680–685.
15. Wang W, Yang T, Li Y, Li S, Yin S, Styles K, Corre C, Yang K. 2016. Development of a Synthetic

Oxytetracycline-Inducible Expression System for Streptomycetes Using de Novo Characterized Genetic Parts. ACS Synth Biol 5:765–773.
16. Li S, Wang J, Li X, Yin S, Wang W, Yang K. 2015. Genome-wide identification and evaluation of constitutive promoters in streptomycetes. Microb Cell Fact 14:172.
17. Okonechnikov K, Golosova O, Fursov M, Varlamov A, Vaskin Y, Efremov I, German Grehov OG, Kandrov D, Rasputin K, Syabro M, Tleukenov T. 2012. Unipro UGENE: A unified bioinformatics toolkit. Bioinformatics.
18. Lemoine F, Correia D, Lefort V, Doppelt-Azeroual O, Mareuil F, Cohen-Boulakia S, Gascuel O. 2019. NGPhylogeny.fr: New generation phylogenetic services for non-specialists. Nucleic Acids Res 47:W260–W265.
19. Dereeper A, Guignon V, Blanc G, Audic S, Buffet S, Chevenet F, Dufayard JF, Guindon S, Lefort V, Lescot M, Claverie JM, Gascuel O. 2008. Phylogeny.fr: robust phylogenetic analysis for the non-specialist. Nucleic Acids Res 36.
20. Letunic I, Bork P. 2021. Interactive Tree Of Life (iTOL) v5: an online tool for phylogenetic tree display and annotation. Nucleic Acids Res 49:W293–W296.
21. Yao MD, Ohtsuka J, Nagata K, Miyazono KI, Zhi Y, Ohnishi Y, Tanokura M. 2013. Complex structure of the DNA-binding domain of AdpA, the global transcription factor in Streptomyces griseus, and a target duplex DNA reveals the structural basis of its tolerant DNA sequence specificity. J Biol Chem 288:31019–31029.
22. Rabyk M, Yushchuk O, Rokytskyy I, Anisimova M, Ostash B. 2018. Genomic Insights into Evolution of AdpA Family Master Regulators of Morphological Differentiation and Secondary Metabolism in Streptomyces. J Mol Evol 86:204–215.
23. Yamazaki H, Tomono A, Ohnishi Y, Horinouchi S. 2004. DNA-binding specificity of AdpA, a transcriptional activator in the A-factor regulatory cascade in Streptomyces griseus. Mol Microbiol 53:555–572.
24. Ohnishi Y, Kameyama S, Onaka H, Horinouchi S. 1999. The A-factor regulatory cascade leading to streptomycin biosynthesis in Streptomyces griseus: Identification of a target gene of the A-factor receptor. Mol Microbiol 34:102–111.
25. Sekurova ON, Zhang J, Kristiansen KA, Zotchev SB. 2016. Activation of chloramphenicol biosynthesis in Streptomyces venezuelae ATCC 10712 by ethanol shock: insights from the promoter fusion studies. Microb Cell Fact 15:85.
