# Supplementary material for: AdpA Positively Regulates Morphological Differentiation and Chloramphenicol Biosynthesis in Streptomyces venezuelae
Source: Microbiol Spectr. 2021 Dec 8;9(3):e01981-21. doi: 10.1128/Spectrum.01981-21 (PMC8653842; doi:10.1128/Spectrum.01981-21)
Supplement: SUPPLEMENTAL FILE 2 — Supplemental material. Download SPECTRUM01981-21_Supp_2_seq7.pdf, PDF file, 2.9 MB [file spectrum01981-21_supp_2_seq7.pdf]

```
                                                      HTH labels and bars omitted
vnz_12630      1 MSQDS-AAP-EAARKLTGRRRREVVAVLLFSGGPIFESSIPLSVFGIDRQDAGVPRYRLLVCAGEDGPLRTTGGLELTAP
SCO2792        1 MSHDSTAAPEAAARKLSGRRRKEIVAVLLFSGGPIFESSIPLSVFGIDRQDAGVPRYRLLVCAGEDGPLRTTGGLELTAP
SGR_4742       1 MSQDS-AAATEAARKLTGRRRREVVAVLLFSGGPIFESSIPLSVFGIDRQDAGVPRYRLLVCGGEEGPLRTTGGLELTAP
SLI_3139       1 MSHDSTAAPEAAARKLSGRRRKEIVAVLLFSGGPIFESSIPLSVFGIDRQDAGVPRYRLLVCAGEDGPLRTTGGLELTAP
SAV_5261       1 MSHDSTAAPDAAARKLSGRRRKEIVAVLLFSGGPIFESSIPLSVFGIDRQDAGVPRYRLLVAAGEEGPLRTTGGLELTAP
SCLAV_1957     1 MSQDS-AAP-EAARKLSGRRRREVVAVLLFSGGPIFESSIPLSVFGIDRQDAGVPRYRLLVCAGEEGPLRTTGGLELTAP
sle_44480      1 MSHDSTAAPDTAARKLSGRRRKEIVAVLLFSGGPIFESSIPLSVFGVDRQDAGVPRYRLLVCAGEEGPLRTTGGLELTAP
consensus      1 **.**.**.. *****.****.*.**********************.**************..**.**************

vnz_12630     79 YGLEAIGRAGTVVVPAWRSITSPPPPEALEALRRAHEEGARIVGLCTGAFVLAAAGLLDGRPATTHWMYAPTLAKRYPSV
SCO2792       81 QGLEAISRAGTVVVPAWRSITSPPPEEALDALRRAHEEGARIVGLCTGAFVLAAAGLLDGRPATTHWMYAPTLAKRYPSV
SGR_4742      80 YGLEAISRAGTVVVPAWRSITSPPPAEALDALRRAHEEGARIVGLCTGAFVLAAAGLLDGRPATTHWMYAPTLAKRYPSV
SLI_3139      81 QGLEAISRAGTVVVPAWRSITSPPPEEALDALRRAHEEGARIVGLCTGAFVLAAAGLLDGRPATTHWMYAPTLAKRYPSV
SAV_5261      81 HGLEAISRAGTVVVPAWRSITSPPPEEALDALRRAHEEGARIVGLCTGAFVLAAAGLLDGRPATTHWMYAPTLAKRYPSV
SCLAV_1957    79 YGLEAIGRAGTVVVPAWRSITSPPPPEALDALRRAHEEGARIVGLCTGAFVLAAAGLLDGRPATTHWMYAPTLAKRYPSV
sle_44480     81 HGLEAISRAGTVVVPAWRSITSPPPQEALDALRRAHEEGARIVGLCTGAFVLAAAGLLDGRPATTHWMYAPTLAKRYPSV
consensus     81  *****.****************** ***.**************************************************

vnz_12630    159 HVDPRELFVDDGDVLTSAGTAAGIDLCLHIVRTDHGTEAAGALARRLVVPPRRSGGQERYLDRSLPEEIGADPLAEVVAW
SCO2792      161 HVDPRELFVDDGDVLTSAGTAAGIDLCLHIVRTDHGNEAAGALARRLVVPPRRSGGQERYLDRSLPEEIGADPLAEVVAW
SGR_4742     160 HVDPRELFVDDGDVLTSAGTAAGIDLCLHIVRTDHGTEAAGALARRLVVPPRRSGGQERYLDRSLPEEIGSDPLAEVVAW
SLI_3139     161 HVDPRELFVDDGDVLTSAGTAAGIPTRPSRTPSDHGNEAAGALARRLVVPPRRSGGQERYLDRSLPEEIGADPLAEVVAW
SAV_5261     161 HVDPRELFVDDGDVLTSAGTAAGIDLCLHIVRTDHGNEAAGALARRLVVPPRRSGGQERYLDRSLPEEIGADPLAEVVAW
SCLAV_1957   159 HVDPRELFVDDGDVLTSAGTAAGIDLCLHIVRTDHGTEAAGALARRLVVPPRRSGGQERYLDRSLPEEIGSDPLAEVVSW
sle_44480    161 HVDPRELFVDDGDVLTSAGTAAGIDLCLHIVRTDHGSEAAGALARRLVVPPRRSGGQERYLDRSLPEEIGADPLAEVVAW
consensus    161 ************************.........***.*********************************.*******.*

                            HTH1                                    HTH2
vnz_12630    239 ALEHLHEQFDVETLAARAYMSRRTFDRRFRSLTGSAPLQWLITQRVLQAQRLLETSDYSVDEVAGRCGFRSPVALRGHFR
SCO2792      241 ALEHLHEQFDVETLAARAYMSRRTFDRRFRSLTGSAPLQWLITQRVLQAQRLLETSDYSVDEVAGRCGFRSPVALRGHFR
SGR_4742     240 ALEHLHEQFDVETLAARAYMSRRTFDRRFRSLTGSAPLQWLITQRVLQAQRLLETSDYSVDEVAGRCGFRSPVALRGHFR
SLI_3139     241 ALEHLHEQFDVETLAARAYMSRRTFDRRFRSLTGSAPLQWLITQRVLQAQRLLETSDYSVDEVAGRCGFRSPVALRGHFR
SAV_5261     241 ALEHLHEQFDVETLAARAYMSRRTFDRRFRSLTGSAPLQWLITQRVLQAQRLLETSDYSVDEVAGRCGFRSPVALRGHFR
SCLAV_1957   239 ALEHLHEQFDVETLAARAYMSRRTFDRRFRSLTGSAPLQWLITQRVLQAQRLLETSDYSVDEVAGRCGFRSPVALRGHFR
sle_44480    241 ALEHLHEQFDVETLAARAYMSRRTFDRRFRSLTGSAPLQWLITQRVLQAQRLLETSDYSVDEVAGRCGFRSPVALRGHFR
consensus    241 ********************************************************************************

vnz_12630    319 RQLGSSPAAYRAAYRARRPQGE--AATAVLDPVVPAQVP---------AGLRRPGSP----LEPGKPQSDAYAP--GRAA
SCO2792      321 RQLGSSPAAYRAAYRARRPQGDRQPDPDTAAAGATRPLPPS-------DPPASLAPENAVPFQTRRTATPMPAGAASVPG
SGR_4742     320 RQLGSSPAAYRAAYRARRPQGVAESAATVVETMVPSQGPPSGRRGSTLSSAAVAVAASVGSGELSLPGPDAYVP--GRPA
SLI_3139     321 RQLGSSPAAYRAAYRARRPQGDRQPDPDTAAAGASRPLPPS-------DPPASLAPENAVPFQTRRTATPMPAGAASVPG
SAV_5261     321 RQLGSSPAAYRAAYRARRPQGERPVDPDSVVGPPPALPQEAPVPMQTRRTAAASSLGPAASLSTEPGKHGSHGSHGSHGS
SCLAV_1957   319 RQLGSSPAAYRAAYRVRRPQGDS-APASLVDSVVPVQARR------TAAAAAGPGGPSSVPPELGKPPSGAYAAGHGRPS
sle_44480    321 RQLGSSPAAYRAAYRARRPQGDRQGDGEGGAATASAATAAGGQTPSAGPPPGPLPADGGVPYQSRRMPS-GPVGSPSTHS
consensus    321 ***************.*****..  . . . .  .   .            .  .   ...  . .  . . ..  ....

vnz_12630    382 LPGQRSAP------------------
SCO2792      394 QRSAP---------------------
SGR_4742     398 LPGQRSAP------------------
SLI_3139     394 QRSAP---------------------
SAV_5261     401 HGSHGSHGSELYAAGRPSLPGQRSAP
SCLAV_1957   392 LPGQRSAP------------------
sle_44480    400 LAAASSDNGREYVPSRASVPGQRSAT
consensus    401 . .  .
```

Fig.S1

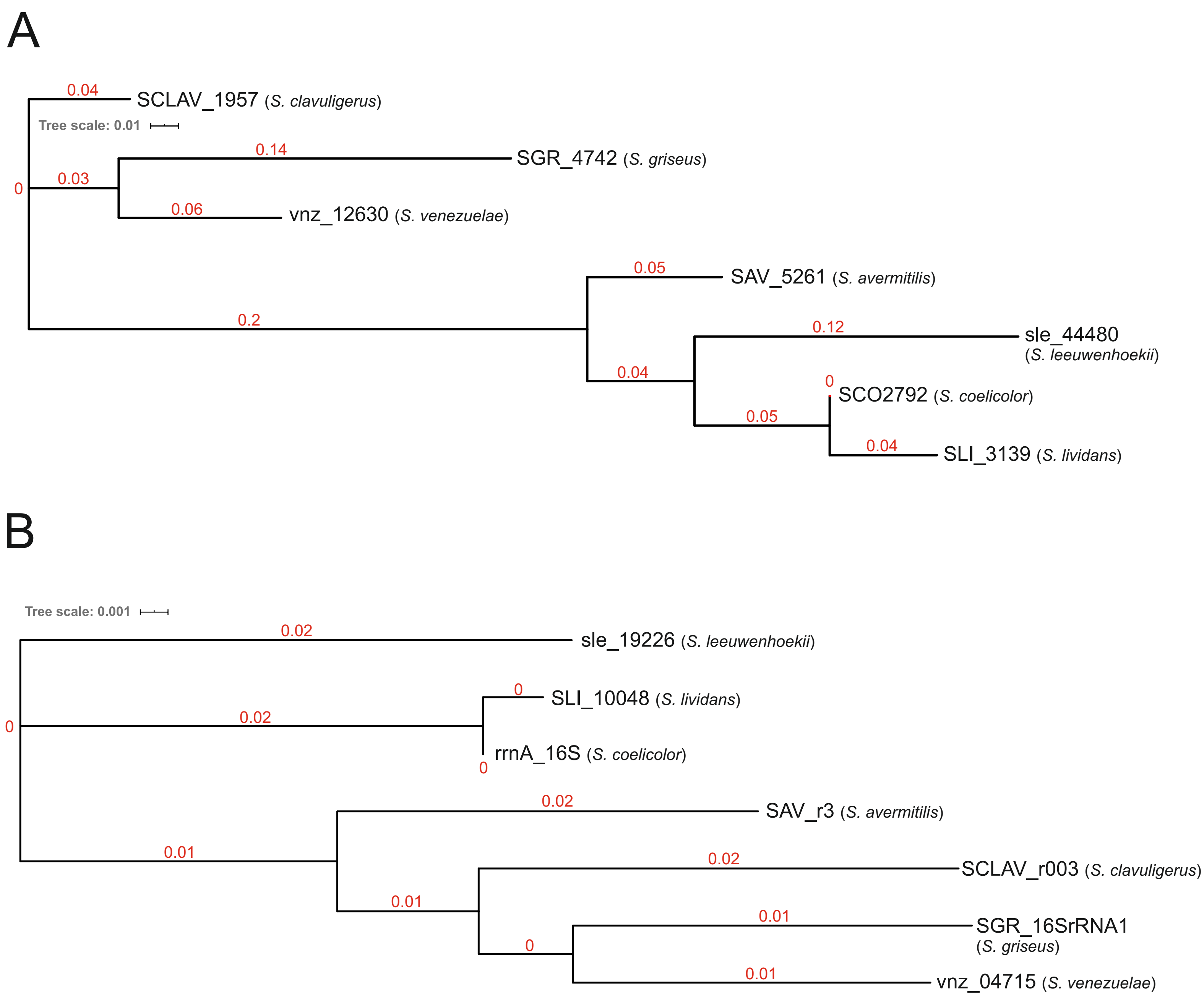

A
0.04
SCLAV_1957 (S. clavuligerus)
Tree scale: 0.01
0.14
SGR_4742 (S. griseus)
0
0.03
0.06
vnz_12630 (S. venezuelae)
0.05
SAV_5261 (S. avermitilis)
0.2
0.12
sle_44480 (S. leeuwenhoekii)
0.04
0
SCO2792 (S. coelicolor)
0.05
0.04
SLI_3139 (S. lividans)
B
Tree scale: 0.001
0.02
sle_19226 (S. leeuwenhoekii)
0
SLI_10048 (S. lividans)
0
0.02
rrnA_16S (S. coelicolor)
0
0.02
SAV_r3 (S. avermitilis)
0.01
0.02
SCLAV_r003 (S. clavuligerus)
0.01
0.01
SGR_16SrRNA1 (S. griseus)
0
0.01
vnz_04715 (S. venezuelae)

Fig.S2

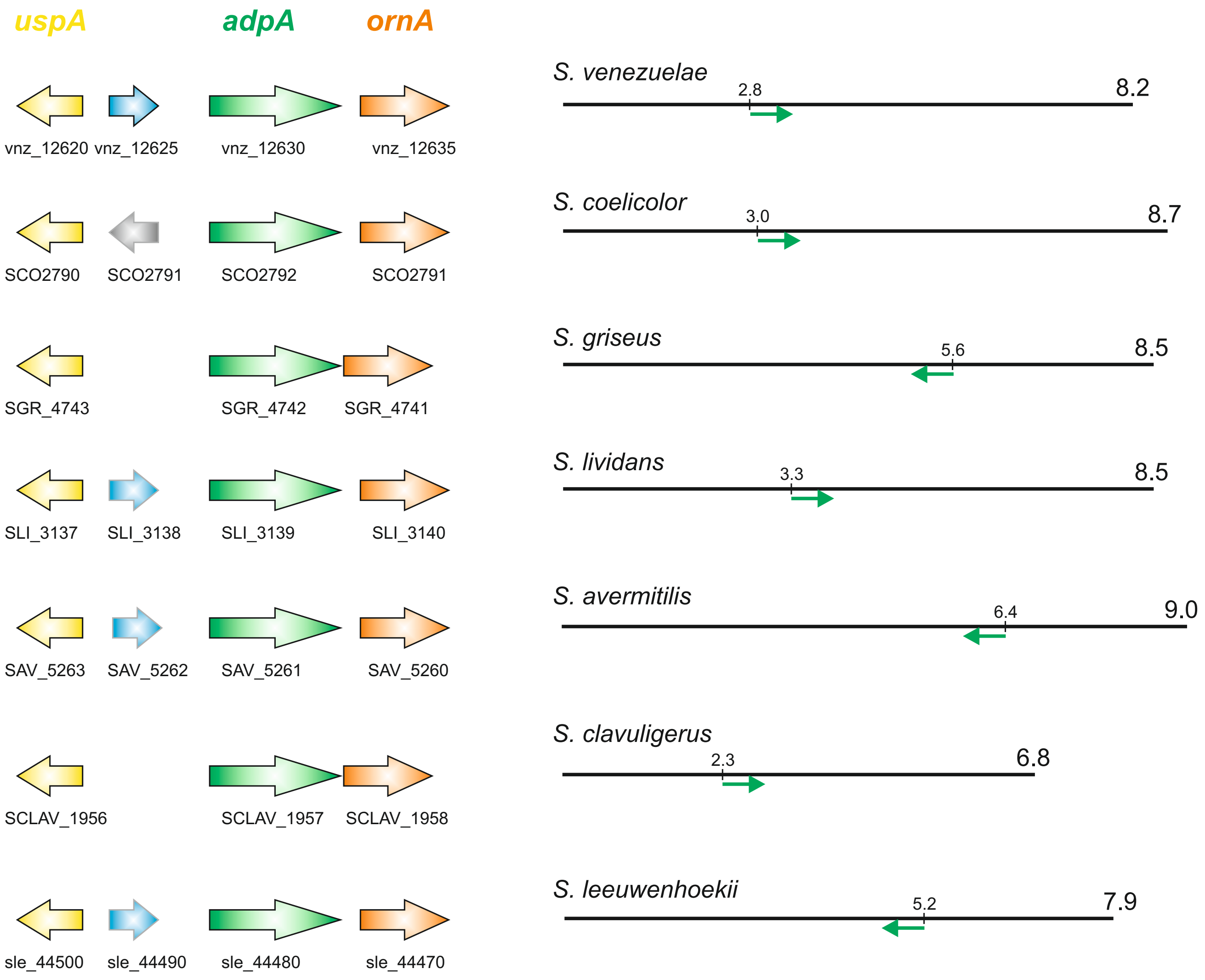
uspA
adpA
ornA
vnz_12620
vnz_12625
vnz_12630
vnz_12635
SCO2790
SCO2791
SCO2792
SCO2791
SGR_4743
SGR_4742
SGR_4741
SLI_3137
SLI_3138
SLI_3139
SLI_3140
SAV_5263
SAV_5262
SAV_5261
SAV_5260
SCLAV_1956
SCLAV_1957
SCLAV_1958
sle_44500
sle_44490
sle_44480
sle_44470
S. venezuelae
2.8
8.2
S. coelicolor
3.0
8.7
S. griseus
5.6
8.5
S. lividans
3.3
8.5
S. avermitilis
6.4
9.0
S. clavuligerus
2.3
6.8
S. leeuwenhoekii
5.2
7.9

Fig.S3

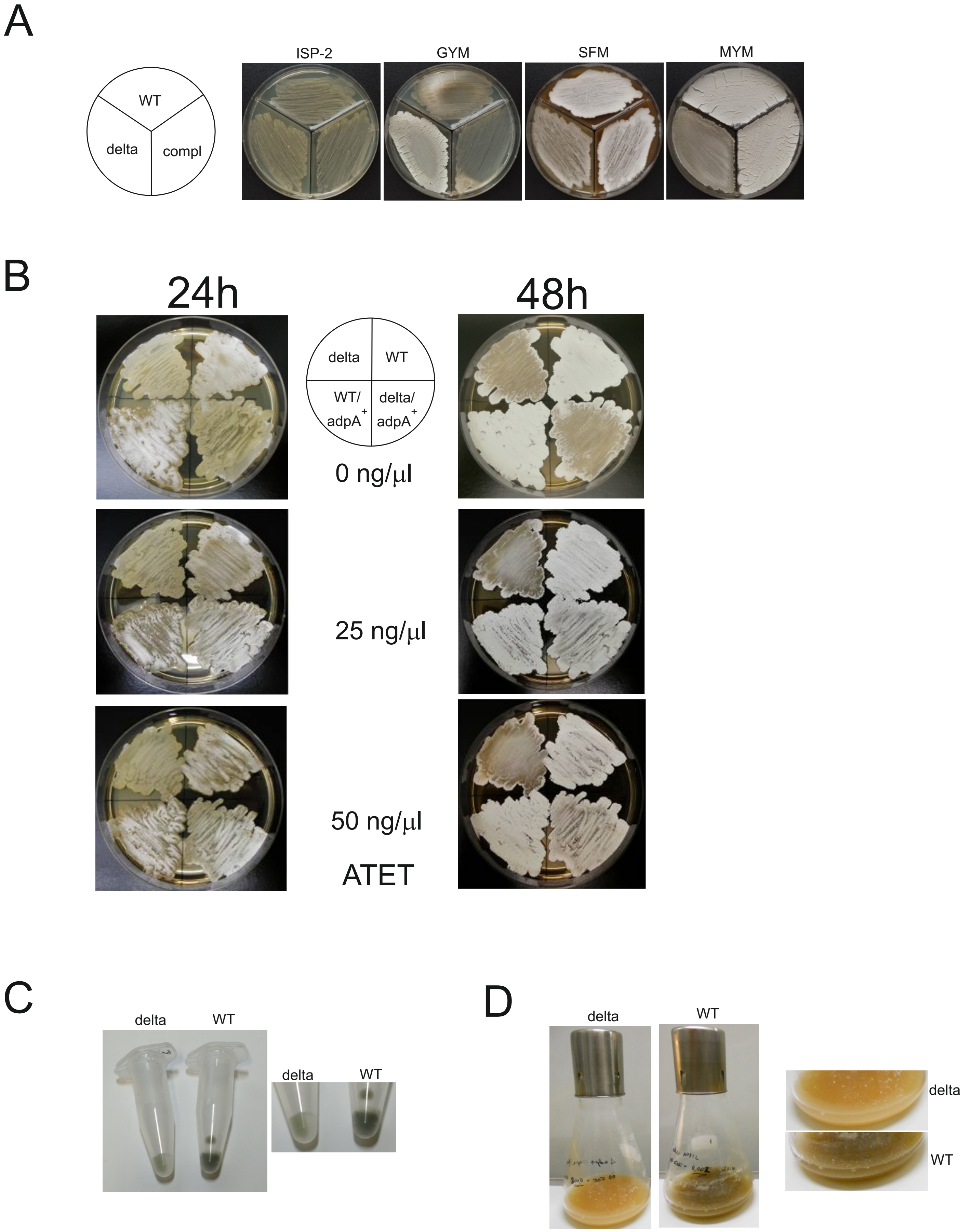
A
WT
delta
compl
ISP-2
GYM
SFM
MYM
B
24h
48h
delta
WT
WT/ adpA+
delta/ adpA+
0 ng/µl
25 ng/µl
50 ng/µl
ATET
C
delta
WT
delta
WT
D
delta
WT
delta
WT

Fig.S4

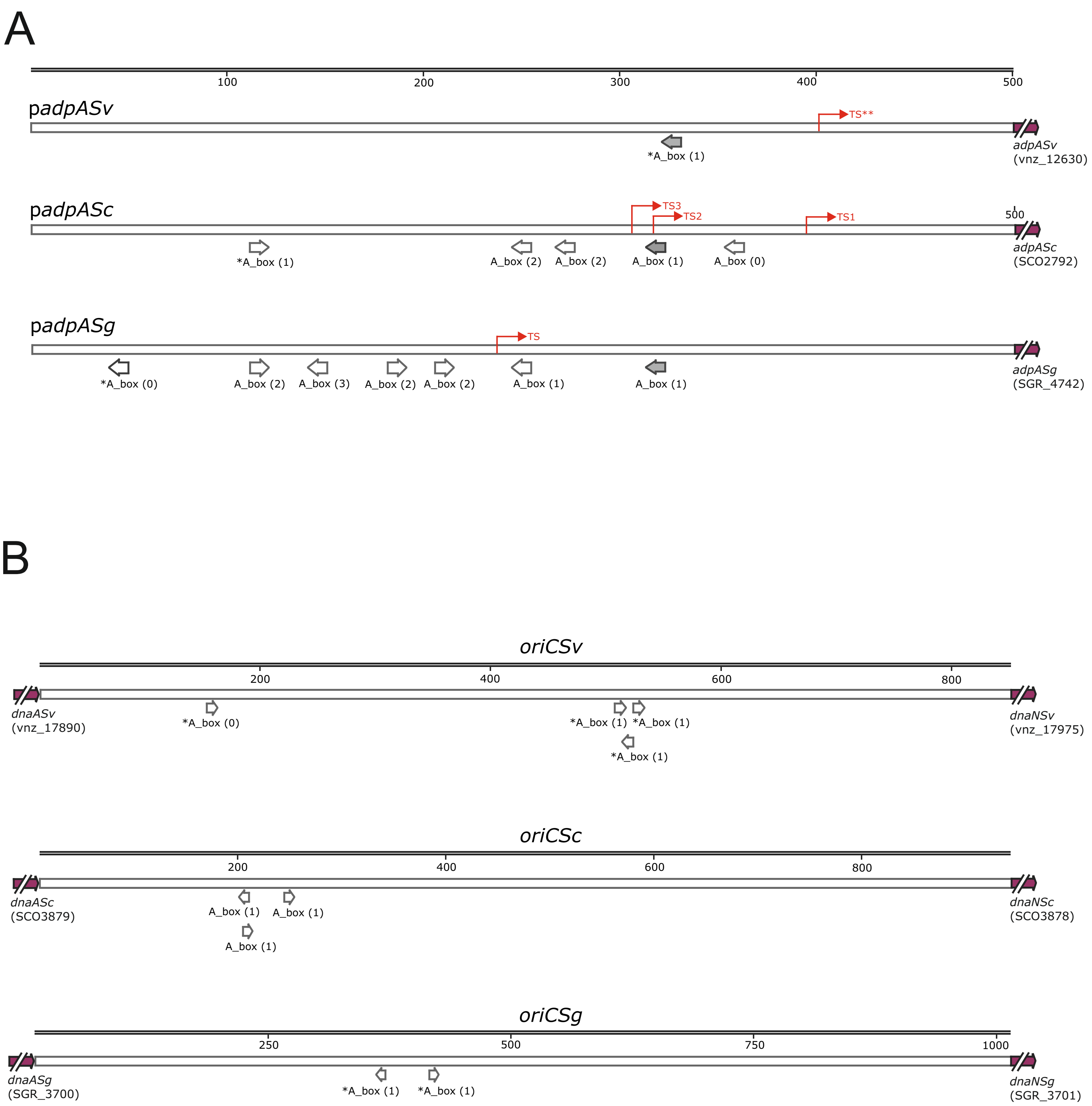

A
100
200
300
400
500
padpASv
TS**
*A_box (1)
adpASv
(vnz_12630)
padpASc
TS3
TS2
TS1
500
*A_box (1)
A_box (2)
A_box (2)
A_box (1)
A_box (0)
adpASc
(SCO2792)
padpASg
TS
*A_box (0)
A_box (2)
A_box (3)
A_box (2)
A_box (2)
A_box (1)
A_box (1)
adpASg
(SGR_4742)
B
oriCSv
200
400
600
800
dnaASv
(vnz_17890)
*A_box (0)
*A_box (1)
*A_box (1)
*A_box (1)
dnaNSv
(vnz_17975)
oriCSc
200
400
600
800
dnaASc
(SCO3879)
A_box (1)
A_box (1)
A_box (1)
dnaNSc
(SCO3878)
oriCSg
250
500
750
1000
dnaASg
(SGR_3700)
*A_box (1)
*A_box (1)
dnaNSg
(SGR_3701)

Fig.S5

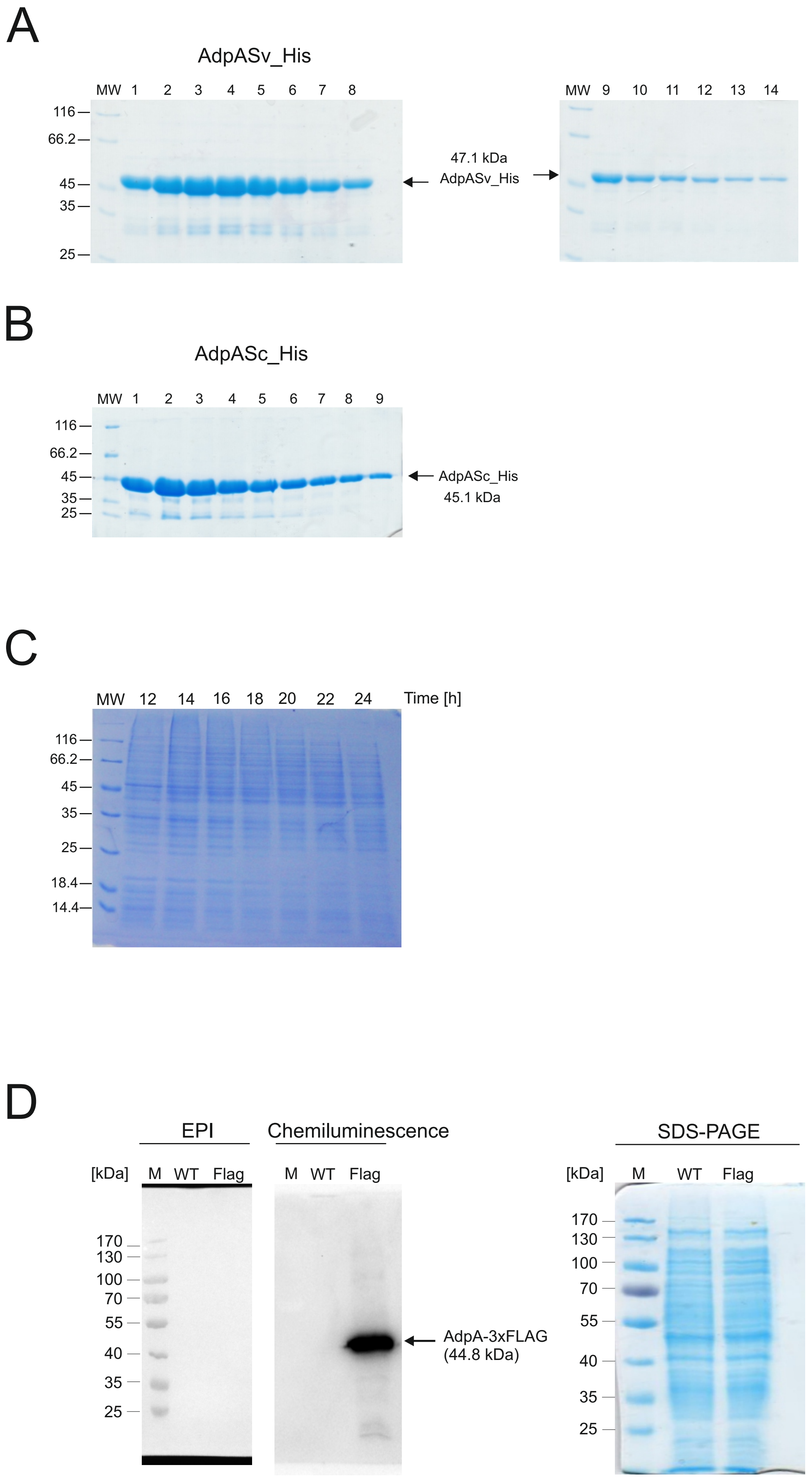
A
AdpASv_His
MW 1 2 3 4 5 6 7 8
116
66.2
45
35
25
47.1 kDa
AdpASv_His
MW 9 10 11 12 13 14
B
AdpASc_His
MW 1 2 3 4 5 6 7 8 9
116
66.2
45
35
25
AdpASc_His
45.1 kDa
C
MW 12 14 16 18 20 22 24 Time [h]
116
66.2
45
35
25
18.4
14.4
D
EPI
Chemiluminescence
SDS-PAGE
[kDa] M WT Flag
M WT Flag
170
130
100
70
55
40
35
25
AdpA-3xFLAG
(44.8 kDa)
[kDa] M WT Flag
170
130
100
70
55
40
35
25

Fig.S6

A

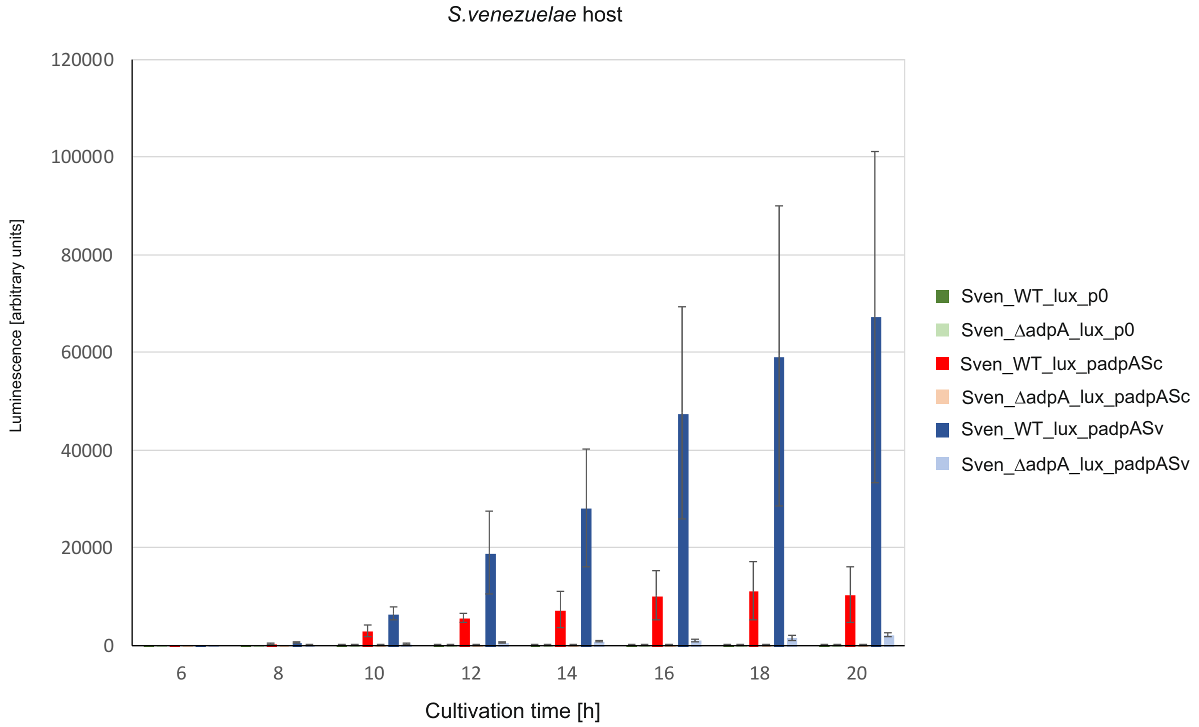

S.venezuelae host
120000
100000
80000
60000
40000
20000
0
Luminescence [arbitrary units]
6
8
10
12
14
16
18
20
Cultivation time [h]
Sven_WT_lux_p0
Sven_ΔadpA_lux_p0
Sven_WT_lux_padpASc
Sven_ΔadpA_lux_padpASc
Sven_WT_lux_padpASv
Sven_ΔadpA_lux_padpASv

B

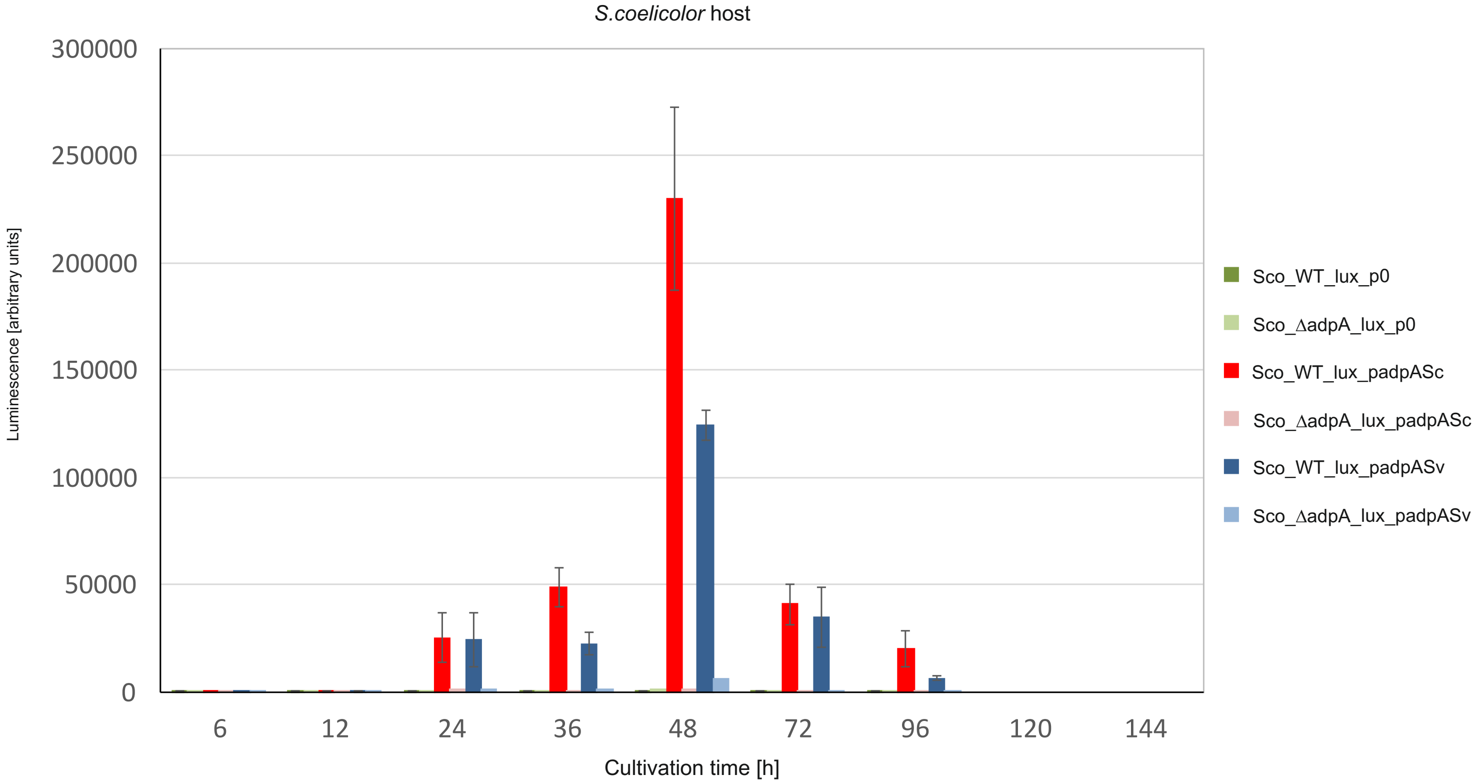

S.coelicolor host
300000
250000
200000
150000
100000
50000
0
Luminescence [arbitrary units]
6
12
24
36
48
72
96
120
144
Cultivation time [h]
Sco_WT_lux_p0
Sco_ΔadpA_lux_p0
Sco_WT_lux_padpASc
Sco_ΔadpA_lux_padpASc
Sco_WT_lux_padpASv
Sco_ΔadpA_lux_padpASv

Fig.S7

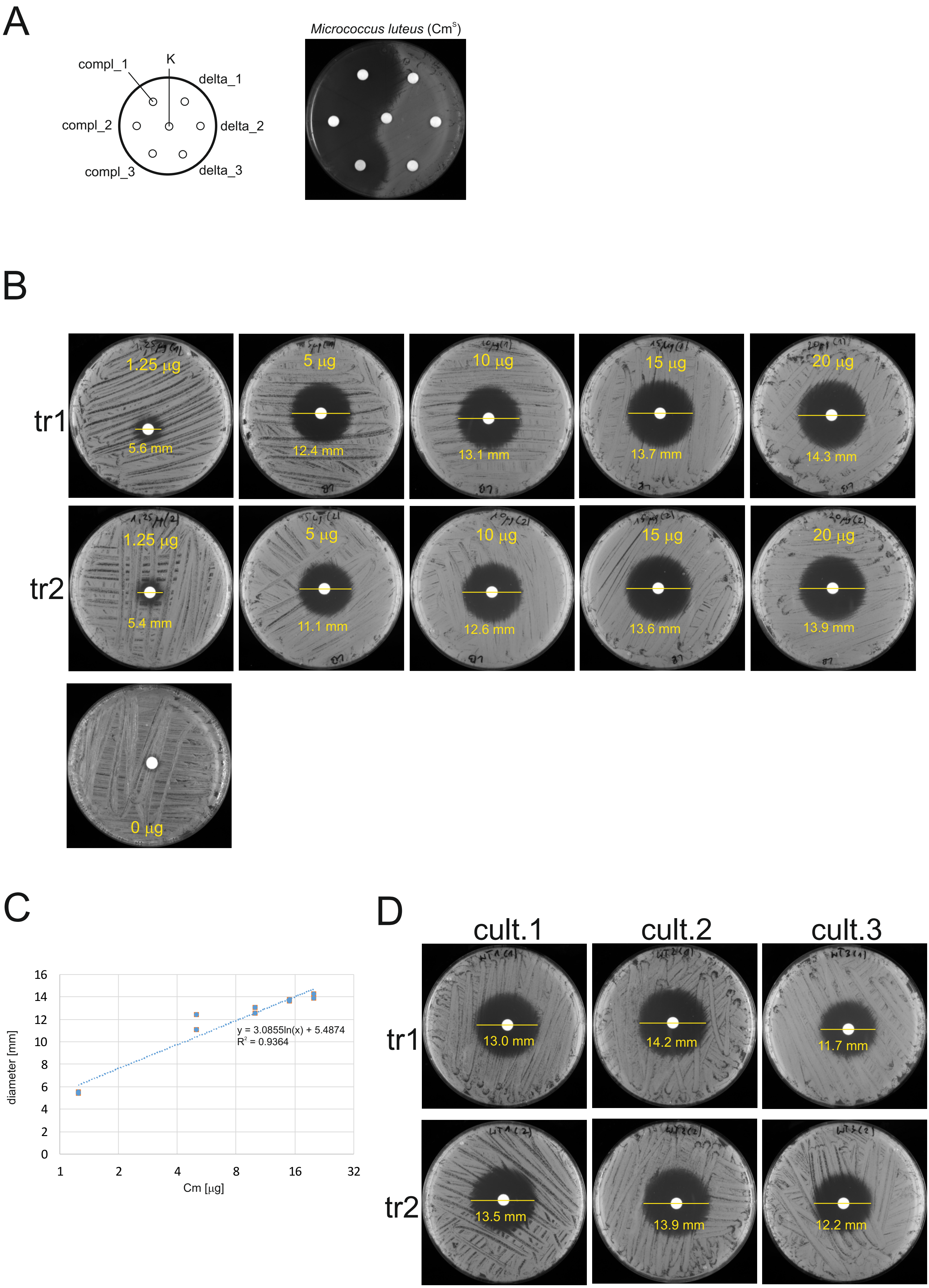
A
compl_1
K
delta_1
compl_2
delta_2
compl_3
delta_3
Micrococcus luteus (Cm^S)
B
tr1
1.25 µg
5.6 mm
5 µg
12.4 mm
10 µg
13.1 mm
15 µg
13.7 mm
20 µg
14.3 mm
tr2
1.25 µg
5.4 mm
5 µg
11.1 mm
10 µg
12.6 mm
15 µg
13.6 mm
20 µg
13.9 mm
0 µg
C
diameter [mm]
Cm [µg]
y = 3.0855ln(x) + 5.4874
R² = 0.9364
D
cult.1
cult.2
cult.3
tr1
13.0 mm
14.2 mm
11.7 mm
tr2
13.5 mm
13.9 mm
12.2 mm

Fig.S8

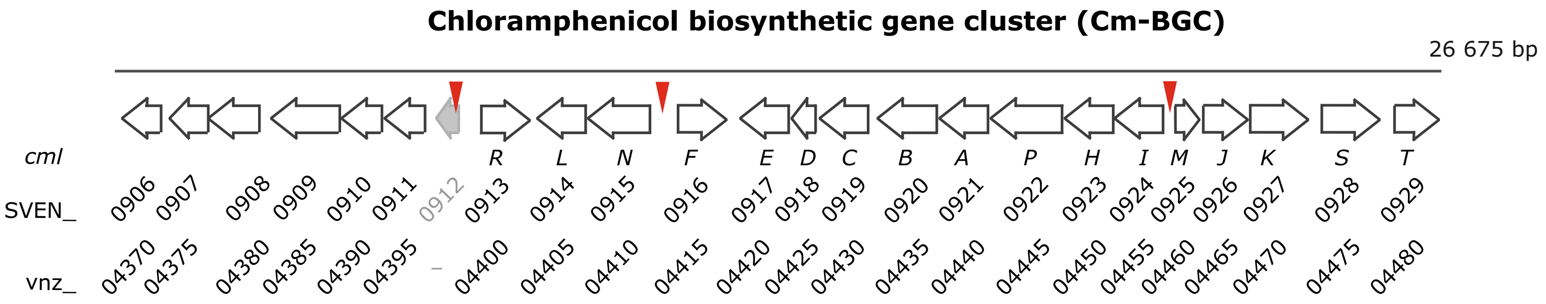
Chloramphenicol biosynthetic gene cluster (Cm-BGC)
26 675 bp
cml
R L N F E D C B A P H I M J K S T
SVEN_
0906 0907 0908 0909 0910 0911 0912 0913 0914 0915 0916 0917 0918 0919 0920 0921 0922 0923 0924 0925 0926 0927 0928 0929
vnz_
04370 04375 04380 04385 04390 04395 – 04400 04405 04410 04415 04420 04425 04430 04435 04440 04445 04450 04455 04460 04465 04470 04475 04480

Fig.S9
